# Editorial: Molecular Pathways Controlling Epithelial Inflammation in the Gut

**DOI:** 10.3389/fimmu.2022.897587

**Published:** 2022-04-25

**Authors:** Yuseok Moon

**Affiliations:** ^1^Laboratory of Mucosal Exposome and Biomodulation, Department of Integrative Biomedical Sciences and Anatomy, Pusan National University, Yangsan, South Korea; ^2^Graduate Program of Genomic Data Sciences, Pusan National University, Yangsan, South Korea

**Keywords:** mucosal immunity, gut microbiota, epithelial barrier, microbial metabolites, mucosal immunoregulation, epithelial inflammation

The gut epithelium is a frontline barrier between luminal contents and the underlying mucosal immune system while facilitating the transport of nutrients and water. Recent scientific advances in the dynamic communications between luminal microbiota and gut epithelial cells (GECs) have greatly increased our understanding of epithelial pathophysiology. Gut microbial exposure triggers various types of pattern-recognition-receptor (PRR)-linked or stress-responsive signaling pathways in GECs. In particular, PRR-activated transcription factors such as NFκB and the interferon-regulatory factor (IRF) play pivotal roles in inducing the genes involved in cellular and humoral immune responses (1–3). Although excessive or persistent PRR-linked signaling activation may lead to detrimental inflammatory cytokine production and subsequent gut barrier disruption, optimally activated epithelial NFκB signals can exert a protective effect by improving cell proliferation in response to tissue injuries. GEC-derived cytokines are also involved in regulating immune responses by mucosa-associated myeloid- or lymphoid-derived cells. In addition to cytokines, GECs can secrete diverse types of defense molecules such as mucins and antimicrobial peptides. All of these components play crucial roles in regulating gut epithelial integrity during infection and tissue injuries. Discordant regulation of the cellular or humoral components in the mucosal immune network would lead to various types of mucosa-associated or systemic distresses, including hypersensitivity and inflammatory outcomes.

In response to the gut microbiota, GECs recognize the structural components of live and dead microbes *via* PRRs. Some PRRs are involved in the recognition of bacteria-derived extracellular vesicles (EVs) in the gut. Martin-Gallausiaux et al. demonstrated that EVs of *Fusobacterium nucleatum*, a pathobiont in the gut lumen, trigger the innate immune responses of intestinal epithelial cells by promoting NFκB signaling in a TLR2-dependent manner, rather than in a TLR4-linked pathway. In particular, the epithelial activation of NFκB signaling was mediated by the non-specific porin FomA, one of the most expressed outer membrane proteins of EVs, which is known to modulate cell adhesion with its immunogenic properties (4). Mechanistically, the transcriptional machinery of NFκB- or IRF-promoted gene expression is tightly regulated by post-translation modifications including ubiquitinylation in immunity-related cells (5, 6). Karhausen et al. addressed the diverse actions of the SUMO-dependent regulation of the interferon or the NFκB signaling pathway in the gut during detrimental and inflammatory stress.

In addition to the bacterial structural components, bacterial metabolites are involved in regulating mucosal immunity. Luminal microbes can metabolize the dietary components and host molecules. For instance, gut bacteria can utilize the dietary fibers by converting them to short-chain fatty acids (SCFAs), which play pivotal roles in regulating host cell differentiation and energy metabolism. Gong et al. suggested that SCFAs such as β-hydroxybutyrate or butyrate activate G protein-coupled receptor 109A (GPR109A, HM74A in humans), which counteracts the proinflammatory signaling cascades of NFκB and inflammasome NLRP3 in response to bacterial infection. Moreover, SCFA-activated GPR109A signaling is involved in inducing polymeric immunoglobulin receptor expression, which facilitates the mucosal secretion of immunoglobulin A and subsequent host defense against bacterial access to the gut epithelial barrier. In addition to GPR109A-mediated immune regulation, SCFAs are involved in the epigenetic control of the gut immune cells *via* the inhibition of HDAC activity. Moreover, some dietary fibers can directly bind to the PRRs, regulating gut immunity. For example, a low degree of methyl esterification pectin inhibits TLR2, whereas β2 fructans activate TLR2-linked signaling.

Gut bacteria-derived tryptophan metabolites are another microbial regulator for epithelial immunity and inflammatory responses. Different bacterial species display different catalytic enzymes, which can generate a typical profile of luminal tryptophan metabolites, including tryptamine, indole-3-pyruvic acid, indole, indole-3-acetaldehyde, indole-lactic-acid, indole-3-acetic acid, tryptophol, skatole, indole-acrylic acid, and indole propionic acid (7). Gasaly et al. compared the differential actions of tryptophan metabolites in the gut barrier. Intestinal epithelial cells recognized these tryptophan metabolites *via* the pregnane X receptor (PXR) or the aryl hydrocarbon receptor (AhR). In particular, indole and indole-3-acetamide, as low- and medium-affinity orthosteric ligands of PXR, regulate intestinal permeability and inflammation in intestinal cells, despite some controversies (8). A variety of indole-based bacterial metabolites are ligands of AhR in the gut epithelial and immune cells. They are mostly involved in maintaining barrier-protective actions during inflammation and hypersensitivity reactions in an AhR-dependent manner. Mechanistically, AhR activation triggers the induction of some members of the IL-10 family, such as IL-10 and IL-22, which play crucial roles in maintaining gut epithelial homeostasis. Generally, IL-10 serves as an important regulator in preventing pro-inflammatory responses, while IL-22 contributes to improving the barrier integrity in response to tissue injury Wei et al., Papoutsopoulou et al. addressed the genetic landscape of IL-10-deficient gut epithelia in response to proinflammatory insults. Intestinal epithelium-derived IL-10 was predicted as a positive regulator of the canonical NFκB pathway, contributing to the maintenance of intestinal homeostasis, particularly during the resolution stage of the inflammation. Although excess levels of NFκB signaling activation are associated with detrimental outcomes during inflammation, the delayed activation of epithelial NFκB may contribute to the reconstitution of injured mucosal monolayers *via* the up-regulation of cell migration-related target genes in response to the tissue injury (9, 10). Therefore, phase-associated signaling responses to gut bacteria are an important readout of disease prognosis in the gut mucosa.

To avoid the detrimental hypersensitivity of the immune response to the luminal antigens, the biological system develops oral tolerance by driving the development of regulatory T cells and regulatory innate lymphoid cells (11). Smaldini et al. demonstrated an immunoregulatory action of Actinomyces *Tsukamurella inchonensis* against food hypersensitivity in an animal model. Mechanistically, the tolerance to the food allergen was associated with the increased action of regulatory T cells and anti-inflammatory cytokines such as IL-10. In addition to the lymphoid cell-mediated immune tolerance, the adult normal intestinal epithelial cells display hypo-responsiveness to commensal bacteria and dietary components (12). At the molecular level, epithelial tolerance to microbiota is mediated by activating the transcription factor peroxisome proliferator-activated receptor γ (PPARγ), a member of the nuclear receptor superfamily abundantly expressed in the gut epithelium (13–15). PPAR-γ-linked signals counteract the NFκB-mediated signaling cascade in gut epithelial cells. The impaired expression of PPAR-γ leads to pathological outcomes in patients with inflammatory bowel diseases, including ulcerative colitis (16). Moreover, Saiz-Gonzalo et al. suggested carcinoembryonic antigen-related cellular adhesion molecules as potent endogenous anti-inflammatory factors of the intestinal epithelial cells during IBD pathogenesis, although there remains a lack of molecular evidence concerning their tolerance.

In summary, the collection in this Research Topic deals with crucial aspects of gut epithelial pathophysiology in association with microbiota, bacterial metabolites, dietary components, and endogenous factors in health and disease (**Figure 1**). This Section highlights the increasing evidence for microbiota- or metabolite-response epithelial signaling contributing to epithelial homeostasis and pathologic processes in the gut.

**Figure 1:**
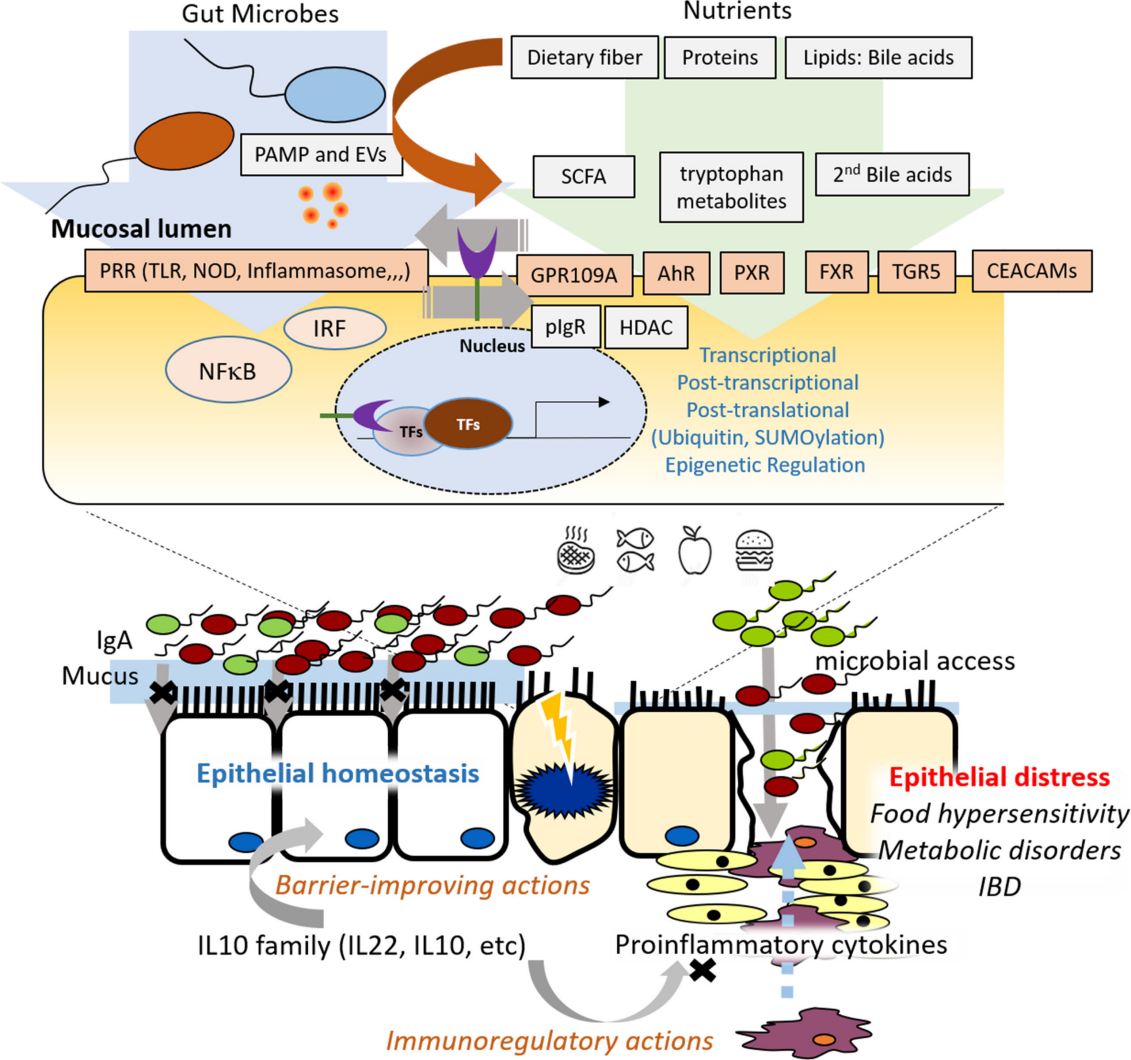
Graphic summary of the research topics on molecular pathways controlling epithelial inflammation in the gut.

## Author Contributions

The manuscript design and hypotheses were defined by YM. YM prepared the manuscript and managed the overall project.

## Funding

This work was supported by a 2-Year Research Grant of Pusan National University.

## Conflict of Interest

The author declares that the research was conducted in the absence of any commercial or financial relationships that could be construed as a potential conflict of interest.

## Publisher’s Note

All claims expressed in this article are solely those of the authors and do not necessarily represent those of their affiliated organizations, or those of the publisher, the editors and the reviewers. Any product that may be evaluated in this article, or claim that may be made by its manufacturer, is not guaranteed or endorsed by the publisher.

## References

[1] Onizawa M Nagaishi T Kanai T Nagano K Oshima S Nemoto Y. Signaling Pathway *via* TNF-Alpha/NF-kappaB in Intestinal Epithelial Cells may be Directly Involved in Colitis-Associated Carcinogenesis. Am J Physiol Gastrointest Liver Physiol (2009) 296(4):G850–9. doi: 10.1152/ajpgi.00071.2008 19179628

[2] Oshima S Nakamura T Namiki S Okada E Tsuchiya K Okamoto R. Interferon Regulatory Factor 1 (IRF-1) and IRF-2 Distinctively Up-Regulate Gene Expression and Production of Interleukin-7 in Human Intestinal Epithelial Cells. Mol Cell Biol (2004) 24(14):6298–310. doi: 10.1128/MCB.24.14.6298-6310.2004 PMC434253 15226432

[3] Wullaert A Bonnet MC Pasparakis M. NF-kappaB in the Regulation of Epithelial Homeostasis and Inflammation. Cell Res (2011) 21(1):146–58. doi: 10.1038/cr.2010.175 PMC3193399 21151201

[4] Nakagaki H Sekine S Terao Y Toe M Tanaka M Ito HO. Fusobacterium Nucleatum Envelope Protein FomA Is Immunogenic and Binds to the Salivary Statherin-Derived Peptide. Infect Immun (2010) 78(3):1185–92. doi: 10.1128/IAI.01224-09 PMC2825909 20008529

[5] Nakagawa K Yokosawa H. Degradation of Transcription Factor IRF-1 by the Ubiquitin-Proteasome Pathway. The C-terminal Region Governs the Protein Stability. Eur J Biochem (2000) 267(6):1680–6. doi: 10.1046/j.1432-1327.2000.01163.x 10712599

[6] Tokunaga F. Linear Ubiquitination-Mediated NF-kappaB Regulation and its Related Disorders. J Biochem (2013) 154(4):313–23. doi: 10.1093/jb/mvt079 23969028

[7] Smith EA Macfarlane GT. Enumeration of Human Colonic Bacteria Producing Phenolic and Indolic Compounds: Effects of Ph, Carbohydrate Availability and Retention Time on Dissimilatory Aromatic Amino Acid Metabolism. J Appl Bacteriol (1996) 81(3):288–302. doi: 10.1111/j.1365-2672.1996.tb04331.x 8810056

[8] Venkatesh M Mukherjee S Wang H Li H Sun K Benechet AP. Symbiotic Bacterial Metabolites Regulate Gastrointestinal Barrier Function *via* the Xenobiotic Sensor PXR and Toll-Like Receptor 4. Immunity (2014) 41(2):296–310. doi: 10.1016/j.immuni.2014.06.014 25065623 PMC4142105

[9] Noiri E Peresleni T Srivastava N Weber P Bahou WF Peunova N. Nitric Oxide is Necessary for a Switch From Stationary to Locomoting Phenotype in Epithelial Cells. Am J Physiol (1996) 270(3 Pt 1):C794–802. doi: 10.1152/ajpcell.1996.270.3.C794 8638659

[10] Cowan MJ Coll T Shelhamer JH. Polyamine-Mediated Reduction in Human Airway Epithelial Migration in Response to Wounding Is PGE2 Dependent Through Decreases in COX-2 and Cpla2 Protein Levels. J Appl Physiol (2006) 101(4):1127–35. doi: 10.1152/japplphysiol.01287.2005 16741257

[11] Newberry RD Hogan SP. Intestinal Epithelial Cells in Tolerance and Allergy to Dietary Antigens. J Allergy Clin Immunol (2021) 147(1):45–8. doi: 10.1016/j.jaci.2020.10.030 33144143

[12] McCole DF Barrett KE. Varied Role of the Gut Epithelium in Mucosal Homeostasis. Curr Opin Gastroenterol (2007) 23(6):647–54. doi: 10.1097/MOG.0b013e3282f0153b 17906442

[13] Mansen A Guardiola-Diaz H Rafter J Branting C Gustafsson JA. Expression of the Peroxisome Proliferator-Activated Receptor (PPAR) in the Mouse Colonic Mucosa. Biochem Biophys Res Commun (1996) 222(3):844–51. doi: 10.1006/bbrc.1996.0832 8651933

[14] Eun CS Han DS Lee SH Paik CH Chung YW Lee J. Attenuation of Colonic Inflammation by PPARgamma in Intestinal Epithelial Cells: Effect on Toll-Like Receptor Pathway. Dig Dis Sci (2006) 51(4):693–7. doi: 10.1007/s10620-006-3193-0 16614990

[15] Kelly D Campbell JI King TP Grant G Jansson EA Coutts AG. Commensal Anaerobic Gut Bacteria Attenuate Inflammation by Regulating Nuclear-Cytoplasmic Shuttling of PPAR-Gamma and RelA. Nat Immunol (2004) 5(1):104–12. doi: 10.1038/ni1018 14691478

[16] Dubuquoy L Jansson EA Deeb S Rakotobe S Karoui M Colombel JF. Impaired Expression of Peroxisome Proliferator-Activated Receptor Gamma in Ulcerative Colitis. Gastroenterology (2003) 124(5):1265–76. doi: 10.1016/S0016-5085(03)00271-3 12730867

